# Visual and auditory attention defects in children with intermittent exotropia

**DOI:** 10.1186/s13052-024-01591-3

**Published:** 2024-01-25

**Authors:** Cong Wei, Ding-Ping Yang, Yan Yang, Wen-Han Yang, Ya-Mei Lu, Xin-Ping Yu, Shuai Chang

**Affiliations:** 1 Zhong Shan Ophthalmological Center, Sun Yat-sen University, Guangzhou, Guangdong China; 2 Qingyuan People’s Hospital, The Sixth Affiliated Hospital of Guangzhou Medical University, Qingyuan, Guangdong China; 3 School of Public Health, Guangdong Pharmaceutical University, Guangzhou, Guangdong China

**Keywords:** Attention defects, Exotropia, Visual, Auditory

## Abstract

**Background:**

Previous studies have shown that children with intermittent exotropia (IXT) have a higher rate of psychiatric abnormalities as they grow up, such as attention deficit. This study explored visual and hearing attention among children with IXT, and evaluated its association with clinical characteristics and cognitive development.

**Methods:**

Forty-nine children with a diagnosis of IXT and 29 children with traditional development were recruited. The Integrated Visual and Auditory Continuous Performance Test (IVA-CPT) was used to measure the subjects’ full-scale response control quotient (FSRCQ), full-scale attention quotient (FSAQ), auditory response control quotient (ARCQ), auditory attention quotient (AAQ), visual response control quotient (VRCQ), and visual attention quotient (VAQ). The Wechsler Intelligence Scale for Children-Fourth Edition (WISC-IV) was used to assess their cognitive function. The differences between the scores of children with IXT and normal controls were analyzed.

**Results:**

The results showed that the FSRCQ, FSAQ, ARCQ, AAQ, VRCQ, and VAQ of children with IXT were all lower than those of normal controls with the same age (*P* < 0.05). The level of attention was significantly correlated with the age of strabismus onset (*P* < 0.05), but not with the degree of strabismus, stereopsis, or fusion control score. In addition, audiovisual attention was correlated significantly with their cognitive development level. The random forest classifier prediction model showed that age of strabismus onset was an important predictor of attention.

**Conclusion:**

Children with IXT have lower visual and auditory attention and control than their peers, and the age of onset of strabismus may be a major factor.

**Supplementary Information:**

The online version contains supplementary material available at 10.1186/s13052-024-01591-3.

## Background

Strabismus is a prevalent ophthalmological disorder. Cases of peripheral origin may arise from ocular trauma, myotonic dystrophy, cranial nerve syndromes, and nerve palsies—such as the reported instance of abducens nerve palsy linked to Sars-Cov-2 infection [1, 2]. A substantial number of cases are concomitant strabismus, which are nonrestrictive and non-paralyzing, originating from possible defects in central neural pathways governing visual perception and kinesthetic control [3, 4]. Intermittent exotropia (IXT) is a subtype of concomitant strabismus, without deficit of exotrocular muscles and nerves, and it is the most common type of strabismus in Asian children, with a prevalence of between 3.24% and 4.69% among Chinese children [5, 6]. An increased incidence of IXT was found among preschool children in a 5-year cohort study [6]. Intermittent exotropia can destroy the patient’s binocular vision function and has a negative impact on some psychosocial functions [7]. Furthermore, a retrospective study found that young adults with IXT onset in childhood were found to be nearly 3 times more likely to develop neuropsychological dysfunction than the general population. Among these subjects, the incidence of attention-deficit/hyperactivity disorder (ADHD) reaches 27–31%, which is much higher than in the general public [8, 9]. Moreover, successful strabismus correction surgery does not seem to reduce the risk of psychiatric disorders in children with IXT [10]. Whether patients with IXT had attention deficits when they were children remains unclear. It is also unclear whether attention deficit is associated with the clinical characteristics of IXT.

Meanwhile, children with exotropia have impaired cognitive ability, including reduced working memory, weakened strategy formation functions, and poor non-verbal performance compared to normal control children [11]. Previous studies suggested that children with exotropia (including IXT) had a different pattern of intelligence structure from the normal control children, with worse perceptual reasoning skills but better processing speeds, while no effects from clinical features were found on the intelligence of children with exotropia [12]. As visual and auditory attention are the prerequisites of cognitive function [13], whether attention deficit contributes to cognitive development in children with IXT remains to be explored.

The integrated visual and auditory-continuous performance test (IVA-CPT) is a commonly used test method in neuropsychology. It quantitatively measures inattention and impulsivity by allowing subjects to complete a mundane automated visual and auditory task, which is a reliable and noninvasive technique [14]. IVA-CPT is often used clinically to diagnose disorders in which attention and control deficits lead to behavioral disorders such as ADHD and post-concussion syndrome [15, 16]. In this study, we tested patients with IXT using IVA-CPT to explore their altered visual and auditory attention, as well as their association with clinical characteristics and cognitive development.

## Methods

### Participants

Children who visited the Zhongshan Ophthalmic Center of Sun Yat-sen University between June 2022 and March 2023 were included in this study. They all voluntarily agreed to take part in the study through recruitment. The inclusion criteria were as follows: (1) age 6–11 years; (2) children with a diagnosis of IXT who had been scheduled for strabismus surgery and an exotropia of more than 15 prism diopters (PD); (3) children with no abnormalities found upon eye examination; (4) 20/25 Snellen acuity or better in each eye with refractive correction. The exclusion criteria were (1) anisometropia (the difference between the spherical lenses of the two eyes > 1.5 D, cylinder > 1.0 D), (2) previous surgery or botulinum injection for strabismus, (3) history of eye disease other than strabismus or refractive error, and (4) history of amblyopia or psychiatric disorders. This study adhered to the Declaration of Helsinki and received approval from the Ethics Committee of the Zhongshan Ophthalmic Center (2022QXPJ004). Before participating in this study, every child and their family provided written or verbal consent after being informed of the details. The age of onset of IXT was reported by the child’s legal caregivers or caregiver. The duration of the IXT refers to the time between the current visit and when it was reported.

### Ophthalmic examinations

All participants underwent a thorough ophthalmic assessment, which included tests for best-corrected visual acuity, slit lamp, intraocular pressure, fundus, and eye movement. The angle of eye deviation was checked at the near (0.33 m) and at a distance (6 m) using the alternate prism cover test. Measurements were performed on at least two different occasions; when necessary, one eye was covered and measured after one hour. The refractive state was detected using cycloplegic (tropicamide) mydriatic optometry. Stereoacuity was tested with Randot circles tests for close range (33 cm) and distance (3 m), and fusional ability was measured using the Worth four-dot test at close range and at a distance. If a child was unable to pass the 400 arcsec level, their stereoacuity was documented as “none” for that particular distance. The Newcastle Control Score (NCS) for office control was introduced to evaluate the maintenance of eye alignment (0 = immediate realignment, 1 = realign with blink or fixation, 2 = remain manifest, 3 = manifest spontaneously).

### The integrated visual and auditory continuous performance test (IVA-CPT)

All participants were tested by professional psychological assessors using IVA-CPT (Braintrain, USA) test software. The examiners did not know whether the child was diagnosed with IXT before the examination. IVA-CPT quotients were used as a result of the examination to reflect the level of attention functioning. The subjects were tested according to a standard examination procedure [17]. Briefly, prior to the test, participants were instructed to practice for 2 to 3 minutes on the computer in order to understand the operation of the test, and the three tasks took about 12 minutes each to complete. During the visual attention test, numbers from 0 to 9 appeared randomly on the screen in the first stage, and the subjects clicked on the number with the mouse whenever “4” appeared. In the second stage, 10 numbers (0–9) appeared on the screen at the same time, and the positions of the numbers were randomly arranged. The subjects looked for and clicked on the number “4.” The third stage was similar to the second stage, but the subjects had to click on the number “7” instead of “4.” During the auditory attention test, the computer randomly played an audio recording of a number (0–9), and when the subjects heard “3,” they clicked on the target on the screen. During the visual and auditory integrated attention test, numbers (0–9) were randomly displayed on the screen, and at the same time, a number was played. When the displayed number was consistent with the played number, the subject clicked on the corresponding number.

The data were recorded automatically during the tests, and 6 comprehensive quotients were generated. Auditory prudence, auditory consistency, and auditory perseverance were reflected in the auditory response control quotient (ARCQ). Visual prudence, visual consistency, and visual perseverance were reflected in the visual response control quotient (VRCQ). The full-scale response control quotient (FSRCQ) was weighted by the ARCQ and VRCQ according to a 1:1 ratio. Auditory alertness, auditory concentration, and auditory speed were reflected in the auditory attention quotient (AAQ). Visual alertness, visual concentration, and visual speed were reflected in the visual attention quotient (VAQ). The full-scale attention quotient (FSAQ) was weighted by AAQ and VAQ according to a 1:1 ratio. The results are presented in standardized coefficients with an average score of 100 and a standard deviation of 15. A score of less than 80 on any of the six indicators was identified as a reduction in attentional functioning. A score of 70 to 80 is classified as a mild abnormality, 61 to 70 as a moderate abnormality, and a score of 60 or less as a severe abnormality [18].

### The wechsler intelligence scale for children–fourth edition (WISC-IV)

The WISC-IV (Wechsler, 2003) is one of the most commonly used instruments to assess cognitive functions in clinical and research settings and has good reliability for use with children and adolescents aged 6 to 16 years [19, 20]. It mainly evaluates the subjects through 10 core tests. Each subtest was included in one of the four main indices and had a standardized mean of 10 with an SD of 3. All 10 sub-tests are combined to get a Full-Scale Intelligence Quotient (FSIQ) score. At the same time, this battery was combined to form four psychologically validated factor scores to describe the levels of different types of cognitive functions. The Verbal Comprehension Index (VCI) includes the Similarities, Vocabulary, and Comprehension subtests; the Perceptual Reasoning Index (PRI) includes the Block Design, Picture Concepts, and Matrix Reasoning subtests; the Working Memory Index (WMI) includes the Digit Span and Letter-Number Sequencing subtests; and the Processing Speed Index (PSI) includes the Coding and Symbol Search subtests.

### Statistical analysis

All collected data were analyzed using SPSS 24.0 statistical software. Continuous variables were described as mean ± standard deviation (SE). When comparing between groups, an independent samples T-test was used for normally distributed data, and the Mann-Whitney U test was used for statistical analysis for non-normal distribution. For enumeration data, the chi-square test was used to compare the differences between the two groups. We used Spearman’s correlation analysis to examine the connection between IVA-CPT and ophthalmic examination indicators. Afterwards, we conducted a linear regression analysis on the indicators that showed statistical significance in the correlation analysis. *P* < 0.05 was statistically significant. We further constructed a random forest classifier prediction model based on individual characteristics and ophthalmic examinations in groups. Meanwhile, feature selection was performed to improve prediction accuracy and decrease the feature dimension by ranking the importance of variables. The importance of individual characteristics and ophthalmic examinations to the prediction model was represented by the Gini coefficient. The higher the Gini coefficient, the higher the predictive value of the parameters for the model. We also generated the multi-dimensional scaling (MDS) plot of the overall participants to intuitively visualize the performance of the prediction model and the difference between the two groups. The random forest model, feature selection process, and MDS plot were all performed using random forest () packages with RStudio software [21].

## Results

### Participants’ demographics and clinical characteristics

The IXT group consisted of 49 children (27 males and 22 females), with a mean age of 7.8 ± 1.3 years, who had been diagnosed with intermittent exotropia (IXT). The control group comprised 29 healthy children (16 male and 13 female) with an average age of 8.1 ± 1.2 years. The two groups of children did not differ significantly in terms of age, gender, or refraction. There was a noticeable difference in visual function between the IXT and CR groups, as indicated in Table 1.

**Table 1:** Demographic and clinical characteristics of the subjects

Variables	IXT (*n* = 49)	CR (*n* = 29)	***P*** value
Male/Femal	27/22	16/13	0.99
Age (years)	7.8 ± 1.3	8.1 ± 1.2	0.32
Age at onset (years)	4.5 ± 3.8	NA	-
Cycloplegic SER (diopters)	-0.28 ± 1.67	+ 0.23 ± 1.31	0.16
Mean eye deviation (prism dioptre)			
Distance	32.1 ± 1.4	0	< 0.001
Near	34.7 ± 1.3	0	< 0.001
Fusional ability (fusion:suppression)			
Distance	24:25	29:0	< 0.001
Near	41:8	29:0	0.02
Stereoacuity (log arcsec)			
Distance Randot	2.9 ± 0.1	1.8 ± 0.2	< 0.001
Near Randot	2.5 ± 0.1	1.9 ± 0.1	< 0.001
NCS for office control			
Distance		NA	-
0	0		
1	1		
2	9		
3	39		
Near		NA	-
0	0		
1	5		
2	21		
3	23		

IXT: intermittent exotropia group, CR: control group, SER: spherical equivalent refraction, NCS: newcastle control score for office, NA: not applicable

### Comparison of IVA-CPT and WISC-IV between the IXT and CR groups

The results of the examination and evaluation of IVA-CPT showed that out of 49 people in the IXT group, 22 had abnormal attention function (44.9%). Six of them with mild abnormalities, 8 with moderate abnormalities, and 8 with severe abnormalities. In contrast, only 5 out of 29 people in the CR group had abnormal attention function (17.2%). Three of them with mildly abnormal, 1 with moderately abnormal, and 1 with severely abnormal. The proportion of abnormal attention was significantly different between the two groups (χ2 = 6.16, *P* = 0.013). All IVA-CPT scores in the IXT group were lower than those in the CR group. Additionally, the FSIQ, VCI, PRI, WMI, and PSI scores were compared between the two groups, with statistically significant differences observed in the FSIQ, VCI, PRI, and PSI scores. Detailed values are summarized in Table 2.

**Table 2:** IVA-CPT and WISC-IV scores of IXT and CR group

	IXT (*n* = 49)	CR (*n* = 29)	***P*** value
IVA-CPT			
FSRCQ	95.76 ± 19.49	107.28 ± 19.64	0.014
FSAQ	93.9 ± 19.14	105.17 ± 19.33	0.014
ARCQ	112.33 ± 19.22	122.66 ± 19.57	0.026
AAQ	110.12 ± 19.28	120.31 ± 19.85	0.029
VRCQ	86.90 ± 17.23	98.90 ± 21.98	0.009
VAQ	85.10 ± 16.95	96.97 ± 21.70	0.009
WISC-IV			
FSIQ	101.92 ± 12.27	114.59 ± 14.98	0.000
VCI	100.90 ± 14.40	112.93 ± 12.90	0.000
PRI	104.57 ± 13.94	115.38 ± 12.08	0.001
WMI	92.96 ± 16.25	99.86 ± 23.09	0.126
PSI	99.88 ± 14.46	108.90 ± 21.89	0.031
Subtests			
Similarities	11.30 ± 3.25	13.41 ± 2.73	0.004
Vocabulary	10.12 ± 3.27	12.31 ± 3.10	0.005
Comprehension	10.10 ± 2.86	10.79 ± 2.30	0.272
Block design	11.84 ± 3.24	14.28 ± 2.66	0.001
Picture concepts	10.08 ± 2.56	11.14 ± 2.86	0.096
Matrix reasoning	10.80 ± 2.86	12.10 ± 2.85	0.054
Digit Span	9.33 ± 2.44	10.03 ± 3.15	0.271
Letter–Number Sequencing	9.33 ± 3.14	11.03 ± 3.39	0.027
Coding	9.98 ± 2.80	11.24 ± 4.61	0.136
Symbol Search	10.37 ± 2.43	11.41 ± 3.70	0.136

Data are presented as the mean ± SD. IXT: intermittent exotropia group; CR: control group; IVA-CPT: integrated visual and auditory continuous performance test; FSRCQ: full-scale response control quotient; FSAQ: Full-scale attention quotient; ARCQ: auditory response control quotient; VRCQ: visual response control quotient; AAQ: auditory attention quotient; VAQ: visual attention quotient; WISC-IV: Wechsler Intelligence Scale for Children–Fourth Edition; FSIQ: full scale intelligence quotient; VCI: verbal comprehension index; PRI: perceptual reasoning index; WMI: working memory index; PSI: processing speed index

### Correlation of IVA-CPT and clinical characteristics

Table 3 shows the correlation between the IVA-CPT scores and the individual characteristics and ophthalmic examinations within the IXT group. The age of onset of intermittent exotropia, degree of exotropia, and fusion ability showed a correlation with IVA-CPT scores. Meanwhile, FSRCQ was correlated with near eye deviation (*r* = 0.302, *P* = 0.038) and distance fusion (*r*=-0.326, *P* = 0.022). FSAQ correlated with near-eye deviation (*r* = 0.303, *P* = 0.037) and distance fusion (*r*=-0.329, *P* = 0.021).

**Table 3:** Correlations of IVA-CPT scores with individual characteristics and ophthalmic examinations

	FSRCQ	FSAQ	ARCQ	AAQ	VRCQ	VAQ
Onset age	0.326*	0.333*	0.322*	0.331*	0.456**	0.458**
Duration	-0.080	-0.078	-0.130	-0.137	-0.120	-0.119
Sex	-0.005	0.003	-0.046	-0.053	-0.241	-0.232
Deviation at Near	0.302*	0.303*	0.193	0.185	0.175	0.181
Deviation at Distance	0.205	0.203	0.167	0.160	0.183	0.184
Fusion at Near	0.060	0.050	0.188	0.185	0.143	0.144
Fusion at Distance	-0.326*	-0.329*	-0.173	-0.177	-0.025	-0.033
Stereoacuity at Near	-0.067	-0.068	-0.076	-0.073	-0.058	-0.057
Stereoacuity at Distance	0.072	0.080	0.115	0.106	-0.141	-0.131
NCS at Near	0.104	0.113	0.039	0.017	0.068	0.073
NCS at Distance	-0.083	-0.071	-0.153	-0.158	0.105	0.109

* *P*＜0.05; ** *P*＜0.01

FSRCQ: full-scale response control quotient; FSAQ: full-scale attention quotient; ARCQ: auditory response control quotient; VRCQ: visual response control quotient; AAQ: auditory attention quotient; VAQ: visual attention quotient; NCS: newcastle control score for office

### Correlation of IVA-CPT and cognitive development

**Fig. 1:**
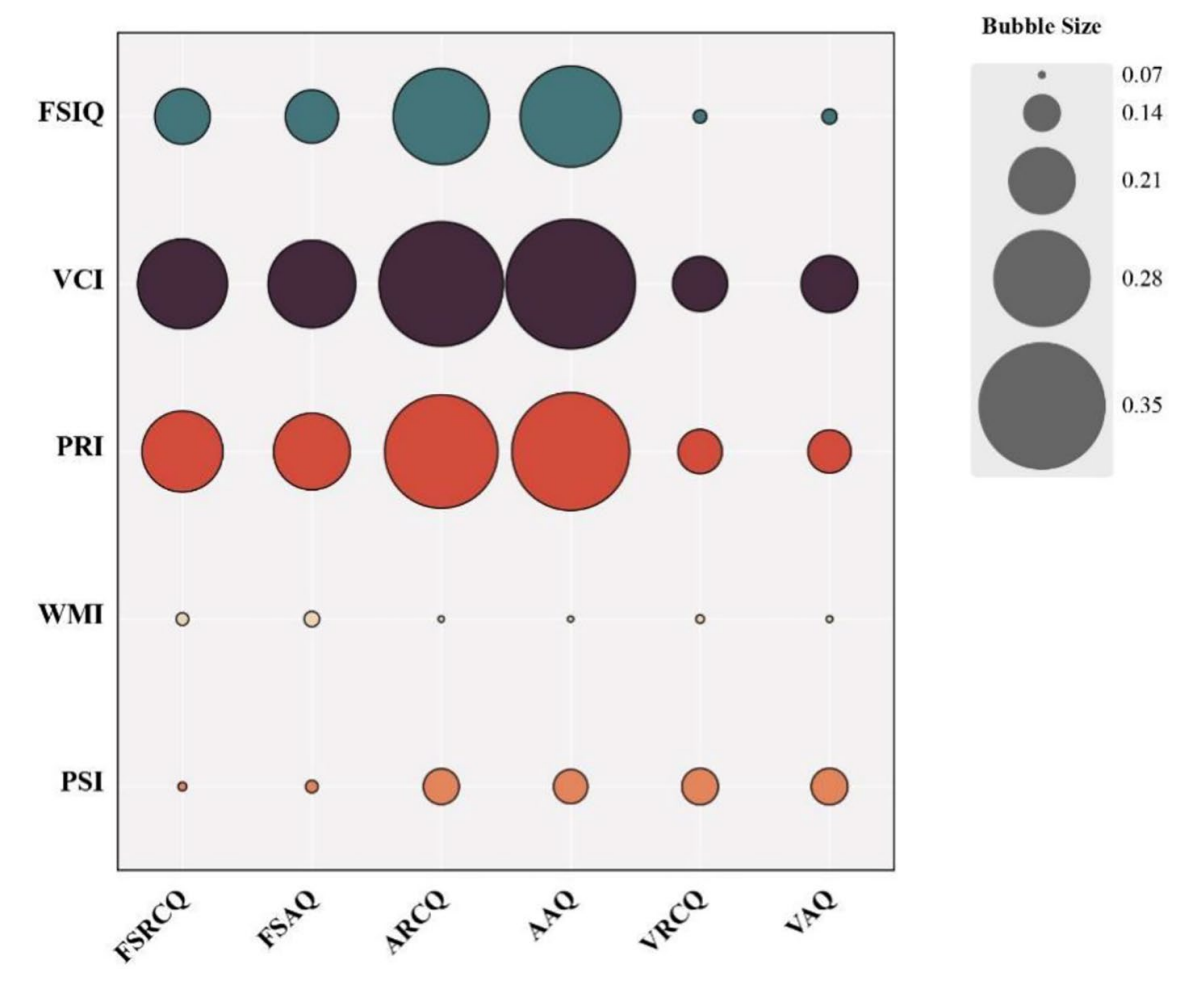
Correlation matrix of six IVA-CPT indicators with WICS scores within the IXT group The bubble size represents the magnitude of the correlation between the two indicators. IXT: intermittent exotropia group; FSRCQ: full-scale response control quotient; FSAQ: full-scale attention quotient; ARCQ: auditory response control quotient; VRCQ: visual response control quotient; AAQ: auditory attention quotient; VAQ: visual attention quotient; FSIQ: full-scale intelligence quotient; VCI: verbal comprehension index; PRI: perceptual reasoning index; WMI: working memory index; PSI: processing speed index

We further analyzed the correlation between IVA-CPT scores and WICS scores, and their correlation matrix is shown in Fig. 1. The bubble size represents the magnitude of the correlation between the two indicators. The ARCQ was correlated with VCI (*r* = 0.343, *P* = 0.005) and PRI (*r* = 0.317, *P* = 0.035). AAQ was correlated with FSIQ (*r* = 0.288, *P* = 0.017), VCI (*r* = 0.355, *P* = 0.004), and PRI (*r* = 0.328, *P* = 0.027), respectively.

### Subgroup analysis of different clinical parameters in the IXT group

We performed a subgroup analysis of the IXT group. Individuals in which IXT occurred before age 4 showed a lower IVA-CPT score than those in which IXT occurred after age four. Interestingly, the 24 individuals with distance fusion had lower FSRCQ and FSAQ scores (89.33 ± 18.66, 87.54 ± 18.29, respectively) than the 25 individuals without distance fusion (101.92 ± 18.58,100.00 ± 18.24, respectively). The difference between the two groups was statistically significant (*P*_FSRCQ_ = 0.022, *P*_FSAQ_=0.021). The IVA-CPT scores were not significantly different from the NCS scores and stereopsis, nor were the deviation angles (Table 4).

**Table 4:** Subgroup analysis of IVA-CPT scores with individual characteristics, ophthalmic examinations in IXT group

Variables	FSRCQ	FSAQ	ARCQ	AAQ	VRCQ	VAQ
Onset Age						
≤4 (*n* = 24)	89.71 ± 21.59	87.79 ± 21.13	108.38 ± 18.73	105.96 ± 18.71	79.75 ± 13.43	78.17 ± 13.27
＞4 (*n* = 25)	101.56 ± 15.54	99.76 ± 15.21	116.12 ± 19.29	114.12 ± 19.34	93.76 ± 17.91	91.76 ± 17.66
*P*	0.032*	0.027*	0.161	0.140	0.003**	0.004**
Deviation at Near						
＜35PD (*n* = 20)	89.70 ± 22.01	88.05 ± 21.78	109.20 ± 17.99	106.95 ± 18.16	85.75 ± 17.00	83.85 ± 16.60
≥35PD (*n* = 29)	99.93 ± 16.68	97.93 ± 16.26	114.48 ± 20.04	112.31 ± 20.03	87.69 ± 17.65	85.97 ± 17.42
*P*	0.070	0.075	0.350	0.344	0.703	0.672
Fusion at Distance						
Fusion (*n* = 24)	89.33 ± 18.66	87.54 ± 18.29	108.96 ± 17.25	106.67 ± 17.43	86.46 ± 17.13	84.54 ± 16.63
Suppression (*n* = 25)	101.92 ± 18.58	100.00 ± 18.24	115.56 ± 20.77	113.44 ± 20.71	87.32 ± 17.68	85.64 ± 17.58
*P*	0.022*	0.021*	0.233	0.223	0.863	0.823
Stereoacuity at Near						
Have (*n* = 20)	94.35 ± 16.67	92.35 ± 16.34	110.10 ± 19.20	108.10 ± 19.64	89.60 ± 18.78	87.55 ± 18.55
None (*n* = 29)	96.72 ± 21.45	94.97 ± 21.06	113.86 ± 19.42	111.52 ± 19.25	85.03 ± 16.6	83.41 ± 15.87
*P*	0.680	0.643	0.506	0.548	0.368	0.407
NCS at Near						
＜3 (*n* = 26)	93.77 ± 19.58	91.84 ± 19.18	111.38 ± 20.15	109.19 ± 20.26	84.23 ± 15.66	82.50 ± 15.42
=3 (*n* = 23)	98.00 ± 19.57	96.22 ± 19.24	113.39 ± 18.50	111.17 ± 18.50	89.91 ± 18.74	88.04 ± 18.43
*P*	0.454	0.431	0.719	0.724	0.254	0.258
NCS at Distance						
＜3 (*n* = 10)	97.50 ± 15.57	95.40 ± 15.02	119.90 ± 13.18	117.40 ± 13.19	81.30 ± 9.08	79.40 ± 8.90
=3 (*n* = 39)	95.31 ± 20.53	93.51 ± 20.21	110.38 ± 20.16	108.26 ± 20.27	88.33 ± 18.58	86.56 ± 18.26
*P*	0.755	0.784	0.165	0.184	0.254	0.237

Data are presented as the mean ± SD;* *P*＜0.05; ** *P*＜0.01

FSRCQ: full-scale response control quotient; FSAQ: full-scale attention quotient; ARCQ: auditory response control quotient; VRCQ: visual response control quotient; AAQ: auditory attention quotient; VAQ: visual attention quotient; NCS: newcastle control score for office; PD: prism diopters

### Random forest classifier prediction model

**Fig. 2:**
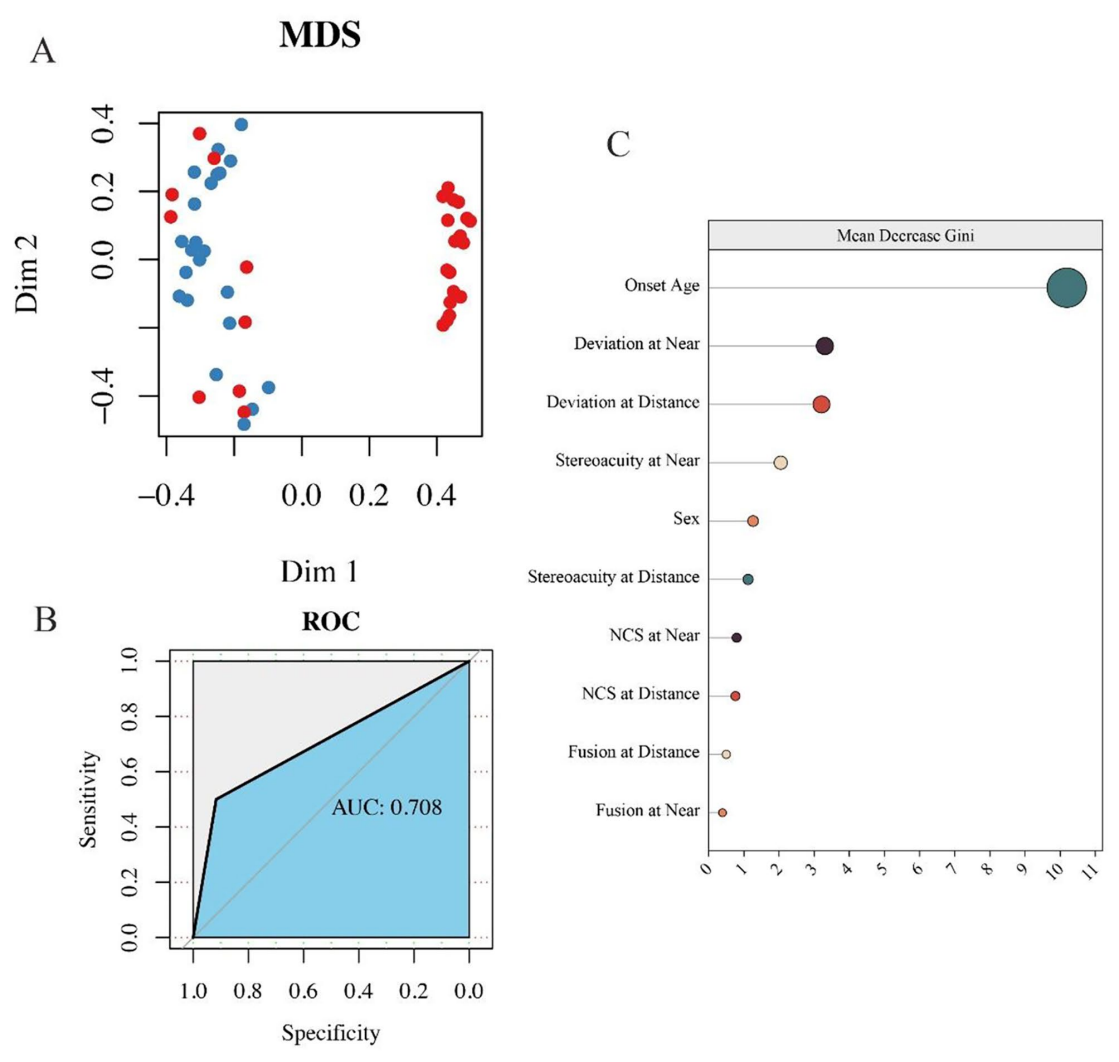
Random forest prediction model to distinguish between IXT and CR groups. **(A)** Multi-dimensional scaling plot visualizing the performance of the random forest prediction model to distinguish between the IXT and CR groups. Red dots indicate subjects with cues for reduced attentional functioning, and blue dots indicate subjects with cues for normal attentional functioning. **(B)** Receiver operator characteristic curve (ROC) describing a random forest prediction model. **(C)** The Gini coefficient generated based on the magnitude of the predictive value of different predictors for the model. NCS stands for Newcastle Control Score for Office

The reliability of the individual characteristics and ophthalmic examinations as reference indicators in the random forest classifier prediction model was assessed on the basis of whether the IVA-CPT examination suggested abnormalities in attentional functioning. The overall predictive effect of the model was expressed as the multidimensional scaling (MDS) plot (Fig. 2A), which shows that the Gini coefficient generated with age at onset as a predictor is significantly higher than the other indicators (Fig. 2C). At the same time, this prediction model has a sensitivity of 0.50, a specificity of 0.91, an accuracy of 0.75 (95% confidence interval 0.51–0.91), a positive predictive value of 0.80, a negative predictive value of 0.73, and a kappa coefficient of 0.44 for the prediction of attentional functioning. Incorporating the predictor into the ROC curve analysis showed that the area under the ROC curve for the predictor was 0.708 (Fig. 2B).

## Discussion

In this study, we found that children with IXT had higher rates of visual and auditory attention deficits compared to their peers. The degree of strabismus, stereopsis function, and fusion control score did not significantly affect attentional abnormality, but the age of onset of strabismus did. Additionally, the study discovered a potential correlation between attention and cognitive function in these children.

Our study revealed that 44.9% of the IXT group had attentional functioning abnormalities and this ratio was about 2.6 times that of the control group. It is consistent with previous literature that patients with strabismus are more likely to suffer from psychiatric disorders in adulthood, in which abnormalities in attentional functioning mainly manifest in ADHD [22, 23]. Previous studies also found that attention deficit occurred more in individuals with IXT: adolescents with IXT occurring in childhood receive about 2.7 times the amount of mental healthcare compared with control [9]. The incidence of ADHD in the adolescents with uncorrected strabismus group was 1.9 times that of the control group, and those in the group with corrected strabismus were about 2.62 times more likely to have ADHD than those in the control group in Israel [24]. Children with strabismus also had a higher risk of hyperactivity/inattention problems, with an OR of 1.64, as reported by their parents [25]. The current study found a higher prevalence of attentional deficits compared to previous studies in children with IXT. This may be due to the fact that these studies were retrospective studies that counted the proportion of children with strabismus who had a record of being clinically diagnosed with ADHD [10, 26], which may underestimate the real prevalence of attentional abnormalities in children with IXT. Another reason may be that the subjects with IXT in this study were children, while the subjects were adolescents or adults in previous studies. Furthermore, although the IVA-CPT we used had definite validity for attention assessment, it is not a diagnostic criterion for ADHD [27]. After previous comparisons in different literatures we found that the level of attention in patients with strabismus differed from that of the normal population, as shown in Table 5, which is consistent with the findings of our current study. In brief, we found that children with IXT have a higher tendency for attention deficit compared with their peers.

**Table 5:** Characteristics of studies related to strabismus and mental disorders

Study	Country	Design	Sample size (Strabismus/Control)	Average age (y) (Strabismus/Control)	No. (%) of patients with mental illness			No. (%) of patients diagnosed with ADHD		
					Strabismus	Control	***P*** value	Strabismus	Control	***P*** value
McKenzie et al. (2009)	United States	Retrospective	183; 183	21.9; 22.2	97 (53.0)	55 (30.1)	< 0.001	28(15.3)	8(4.4)	0.001
Lee et al. (2022)	United States	Cross-sectional	163,439; 11,652,553	8.0; 8.0	1416 (0.9)	60,905 (0.5)	< 0.001	NA	NA	NA
Olson et al. (2012)	United States	Retrospective	127; 127	20.4; 19.1	42 (33.1)	20 (15.7)	0.025	8 (6.3)	6 (4.7)	0.583
Mohney et al. (2008)	United States	Cross-sectional	141; 141	20.3; 20.9	75 (53.2)	38 (27.0)	< 0.001	25(17.7)	7(5.0)	0.001
Our study	China	Cross-sectional	49; 29	7.8; 8.1	NA	NA	NA	22(44.9)	5(17.2)	0.013

ADHD: attention-deficit/hyperactivity disorder; NA: not applicable

A negative correlation was found between attention scores and fusional function in the current study. Children with IXT who had fusion function had lower attention levels, which we speculated may be the reason that IXT patients consume too much attention in the process of controlling eye position and fusing visual information in both eyes. We have also found evidence that children with NCS of 3 had better visual attention and visual control than those with less than 3, though this difference was not statistically significant. Overall, our results suggested that better control of the IXT may be accompanied by poorer visual and hearing attentional ability, while poorer control of IXT may result in better attentional ability. This may involve the allocation of attention resources in subjects with IXT [28, 29]. No significant correlation was found between the level of attention and the degree of eye deviation and stereopsis function. Children with IXT were thought to have a lower health-related quality of life (HRQOL) related to the larger strabismic deviation [30]. The HRQOL assessment also involves a number of attentional state questions (i.e., “It is hard to concentrate because of my eyes.”). Future studies would do well to explore this by extending the range of the degree of strabismus.

Our research also indicates that the level of attention is related to the onset age of IXT provided by caregivers. Specifically, attention issues are more significant when strabismus is detected at an earlier stage, and children who show signs before age four are more likely to experience attention-related issues. There was no significant correlation between the duration of IXT and attention. The random forest model showed that the predictive value of the Gini coefficient generated with age at onset was significantly higher than other indicators. Based on the above discoveries, we assumed that the age at which strabismus becomes noticeable was the most significant factor that correlated with attention development. As is widely accepted, the development of attention begins on the first day of life and is manifested by the responsive movement of the eye to external stimuli [31]. Early attentional orienting functions are developmentally stable at one year of age, and the maintenance of attention is relatively stable at three years of age [32]. We found that children whose IXT symptoms were evident before the age of 4 years were more likely to have accompanying attention deficits. This indicates that the onset of IXT before attention development may significantly affect children’s attention development. Our study also suggests that the earlier the onset of IXT, the more pronounced the attention deficit in children.

Children with IXT were found to have decreased attentional abilities in the study, supported by the visual pathway abnormalities present in patients with strabismus. The human attention system has two orienting networks: one located in the dorsal region, which is responsible for quick strategic eye movements, and another in the ventral area, which responds to incoming stimuli from various sources [33]. The simultaneous transmission of the two pathways plays an important role in the bottom-up arrival of attentional signals to specific areas of the senses [34]. Previous studies on the visual pathway and visual cortex in strabismus also supported the probability mechanism of attention deficit in individuals with IXT: those with strabismus had the volume of gray matter in the occipital lobe and parietal lobe of the brain’s visual cortex decreased, and the activation signal was lower than that of normal people, while the volume of gray matter in areas such as the prefrontal cortex increased [35, 36]. Our finding of reduced both visual attention and auditory attention indicated that the integrated processing of visual and auditory information may also be defective in patients with IXT. Since perception of visual information enhances auditory-related frequency discrimination [37] (e.g., loudness and auditory rhythm perception) [38], the process of integrating vision and hearing can be influenced by binocular vision function (i.e., adults with amblyopia were found to have reduced audiovisual integration) [39].

According to Sun et al., children with exotropia have an atypical pattern of intelligence structure, but their angle of deviation and stereoacuity do not seem to have an impact on their cognitive abilities [12]. Our study showed that children with IXT have lower FSIQ than children of the same age, mainly in terms of reduced verbal comprehension and perceptual reasoning, and reduced processing speed. They performed poorly on the subtests of Similarities, Vocabulary, Block design and Letter-Number Sequencing. Attention is an integral part of the whole process of cognition, from the selection of information to the maintenance of its processing. We also found a correlation between the level of attention and cognitive function in children with IXT. A previous study also showed that alteration in the central brain may cause changes in the level and structure of cognition [11], which is consistent with our findings. Defects in attention found in children with IXT may contribute to the development of cognition.

Although our study found higher rates of visual and auditory attention deficits in children with IXT compared to their peers, the visual and auditory attention levels could be associated with the age of onset of IXT. It is important to note the study’s limitations. First, the IVA-CPT test is an objective measure, but it may be affected by factors such as the time of day or the subject’s mood, potentially leading to abnormal results. At the same time, the results provided a general score on an individual’s level of attention in visual and auditory domain. Second, our sample size was relatively small, and the follow-up period was brief. Continuous monitoring of the changes in attention functions in children with IXT after surgery is necessary. Third, our findings are only a preliminary exploration of the link between IXT and reduced visual and auditory attention in childhood. It is important to assess which specific part in the cognitive process are delayed or distorted in strabismic children. Further research is needed to fully understand this connection.

## Conclusion

In conclusion, our study found that patients with intermittent exotropia show reduced visual and auditory attention in childhood. This reduction does not correlate significantly with the degree of strabismus, fusion control score, or stereopsis function, but rather with the age of onset of strabismus. Our study also suggests that attention deficits may contribute to cognitive development in children with IXT.

### Electronic supplementary material

Below is the link to the electronic supplementary material.

Supplementary Material 1

## Data Availability

No data are available.

## Abbreviations

IXT: Intermittent exotropia
IVA-CPT: Integrated Visual and Auditory Continuous Performance Test
FSRCQ: full-scale response control quotient
FSAQ: full-scale attention quotient
ARCQ: auditory response control quotient
AAQ: auditory attention quotient
VRCQ: visual response control quotient
VAQ: visual attention quotient
WISC-IV: Wechsler Intelligence Scale for Children-Fourth Edition
FSIQ: full-scale intelligence quotient
VCI: verbal comprehension index
PRI: perceptual reasoning index
WMI: working memory index
PSI: processing speed index
ADHD: attention-deficit/hyperactivity disorder
