# Supplementary material for: Visual and auditory attention defects in children with intermittent exotropia
Source: Ital J Pediatr. 2024 Jan 25;50:17. doi: 10.1186/s13052-024-01591-3 (PMC10809432; doi:10.1186/s13052-024-01591-3)
Supplement: Supplementary file 1 — Supplementary Material 1 [file 13052_2024_1591_MOESM1_ESM.docx]

**Response to reviewers**

On behalf of all the contributing authors, I would like to express our sincere appreciation of your letter and the reviewers’ constructive comments concerning our article entitled **“Visual and Auditory Attention Defects in Children with Intermittent Exotropia”.** According to the editor and the reviewers’ comments, we have made extensive modifications to our manuscript and figures to make our results convincing. Point-by-point responses to the nice editor and the three nice reviewers are listed below this letter.

If there are any other modifications we could make, we would like very much to modify them and we really appreciate your help.

**Reviewer 1**

I congratulate you on the chosen topic of undoubted pediatric interest and perhaps not yet sufficiently/duly explored. The work was carried out with a correct and well explained methodology. I would deepen the background by adding a classification of the various types of strabismus in pediatric age based on the etiology and type, also including the most recent cases linked to Sars-Cov-2 infection (Capponi M, Cinicola BL, Brindisi G, Guido CA, Torcé MC, Zicari AM, Spalice A. COVID-19 and abducens nerve palsy in a 9-year-old girl-case report. Ital J Pediatr. 2022 Jun 18;48(1):102. doi: 10.1186‬/s13052-022-01298-3‬. PMID: 35717391‬; PMCID: PMC9206130). The discussion is complete and the conclusions are clear.‬‬‬‬‬‬‬‬‬‬‬‬

Response: We feel great thanks for your professional review work on our article. According to your nice suggestions, we have added information about the classification of strabismus in the background section of the study and cited the most recent cases linked to Sars-Cov-2 infection. The revised sentences are as follows:

“Strabismus is a prevalent ophthalmological disorder. Cases of peripheral origin may arise from ocular trauma, myotonic dystrophy, cranial nerve syndromes, and nerve palsies—such as the reported instance of abducens nerve palsy linked to Sars-Cov-2 infection. A substantial number of cases are concomitant strabismus, which are nonrestrictive and non-paralyzing, originating from possible defects in central neural pathways governing visual perception and kinesthetic control.” **(Lines 53 to 58)**

Reviewer 2

The article offers an important contribution on the subject. However, it would be necessary to include in the discussion more details about the comparison between the results obtained and what is found in the literature, possibly including a table to make the results clearer.

Response: Thank you for pointing this out. We have summarized and compared the relevant studies with the present study, and the relevant results we showed in Table 5, which makes our study clearer. Thanks for your suggestions.

After previous comparisons in different literatures we found that the level of attention in patients with strabismus differed from that of the normal population, as shown in Table 5, which is consistent with the findings of our current study. **(Lines 274 to 277)**

| **Table 5.** Characteristics of studies related to strabismus and mental disorders | | | | | | | | | | |
| --- | --- | --- | --- | --- | --- | --- | --- | --- | --- | --- |
| Study | Country | Design | Sample size (Strabismus/Control) | Average age (y) (Strabismus/Control) | No. (%) of patients with mental illness | |  | No. (%) of patients diagnosed with ADHD | |  |
|  |  |  |  |  | Strabismus | Control | *P* value | Strabismus | Control | *P* value |
| McKenzie et al. (2009) | United States | Retrospective | 183; 183 | 21.9; 22.2 | 97 (53.0) | 55 (30.1) | <.001 | 28（15.3） | 8（4.4） | 0.001 |
| Lee et al. (2022) | United States | Cross-sectional | 352,636; 11,652,553 | 8.0; 8.0 | 1416 (0.9) | 60905 (0.5) | <.001 | NA | NA | NA |
| Olson et al. (2012) | United States | Retrospective | 127; 127 | 20.4; 19.1 | 42 (33.1) | 20 (15.7) | 0.025 | 8 (6.3) | 6 (4.7) | 0.583 |
| Mohney et al. (2008) | United States | Cross-sectional | 141; 141 | 20.3; 20.9 | 75 (53.2) | 38 (27.0) | <.001 | 25(17.7) | 7(5.0) | 0.001 |
| Our study | China | Cross-sectional | 49; 29 | 7.8; 8.1 | NA | NA | NA | 22(44.9) | 5(17.2) | 0.013 |

ADHD, attention-deficit/hyperactivity disorder; NA, not applicable.

Reviewer 3

Thank you for choosing me as a reviewer, following are the reviews from my end -

1. The severity / levels of ADHD (Mild/Moderate /Severe) should be mentioned (which level of children were assessed?)

Response: Thanks to your wonderful advice, we assessed the visual and hearing attention in children with intermittent exotropia (IXT) and compared with controls, in order to explore the visual and hearing attention among children with IXT. Now we have added the criteria for evaluating the results of the IVA-CPT and compared our results in different degrees according to your suggestions. Thank you for pointing this out.

A score of 70 to 80 is classified as a mild abnormality, 61 to 70 as a moderate abnormality, and a score of 60 or less as a severe abnormality. **(Lines 155 to 156)**

2. Areas of cognitive deficits should be specifically mentioned eg. working memory consists of several domains, so what domains are targeted in study. terms used are more generalised.

Response: We feel great thanks for your professional review work on our article. According to your suggestions, we have analyzed the subscales of the WISC-IV scores in more detail to look for more specific causes of cognitive deficits. The relevant results are added to Table 2, and your suggestions are much appreciated.

Each subtest was included in one of the four main indices and had a standardized mean of 10 with an SD of 3. **(Lines 161 to 162)**

Our study showed that children with IXT have lower FSIQ than children of the same age, mainly in terms of reduced verbal comprehension and perceptual reasoning, and reduced processing speed. They performed poorly on the subtests of Similarities, Vocabulary, Block design and Letter-Number Sequencing. **(Lines 332 to 336)**

| **Table 2.** IVA-CPT and WISC-IV scores of IXT and CR group | | | |
| --- | --- | --- | --- |
|  | **IXT (n=49)** | **CR (n=29)** | ***P* value** |
| IVA-CPT |  |  |  |
| FSRCQ | 95.76±19.49 | 107.28±19.64 | 0.014 |
| FSAQ | 93.9±19.14 | 105.17±19.33 | 0.014 |
| ARCQ | 112.33±19.22 | 122.66±19.57 | 0.026 |
| AAQ | 110.12±19.28 | 120.31±19.85 | 0.029 |
| VRCQ | 86.90±17.23 | 98.90±21.98 | 0.009 |
| VAQ | 85.10±16.95 | 96.97±21.70 | 0.009 |
| WISC-IV |  |  |  |
| FSIQ | 101.92±12.27 | 114.59±14.98 | 0.000 |
| VCI | 100.90±14.40 | 112.93±12.90 | 0.000 |
| PRI | 104.57±13.94 | 115.38±12.08 | 0.001 |
| WMI | 92.96±16.25 | 99.86±23.09 | 0.126 |
| PSI | 99.88±14.46 | 108.90±21.89 | 0.031 |
| Subtests |  |  |  |
| Similarities | 11.30±3.25 | 13.41±2.73 | 0.004 |
| Vocabulary | 10.12±3.27 | 12.31±3.10 | 0.005 |
| Comprehension | 10.10±2.86 | 10.79±2.30 | 0.272 |
| Block design | 11.84±3.24 | 14.28±2.66 | 0.001 |
| Picture concepts | 10.08±2.56 | 11.14±2.86 | 0.096 |
| Matrix reasoning | 10.80±2.86 | 12.10±2.85 | 0.054 |
| Digit Span | 9.33±2.44 | 10.03±3.15 | 0.271 |
| Letter–Number Sequencing | 9.33±3.14 | 11.03±3.39 | 0.027 |
| Coding | 9.98±2.80 | 11.24±4.61 | 0.136 |
| Symbol Search | 10.37±2.43 | 11.41±3.70 | 0.136 |
| Data are presented as the means±SD IXT intermittent exotropia group, CR control group, IVA-CPT integrated visual and auditory continuous performance test, FSRCQ full-scale response control quotient, FSAQ Full-scale attention quotient, ARCQ auditory response control quotient, VRCQ visual response control quotient, AAQ auditory attention quotient, VAQ visual attention quotient, WISC-IV Wechsler Intelligence Scale for Children–Fourth Edition, FSIQ full scale intelligence quotient, VCI verbal comprehension index, PRI perceptual reasoning index, WMI working memory index, PSI processing speed index. | | | |

3. Which parts of auditory or visual cognition / cycle eg. perception or comprehension or closure etc are specially delayed or distorted?

Response: Thanks for the question. We agree that it is important to assess which specific part in the cognitive process are delayed or distorted in strabismic children. However, based on our current results, we may not provide evidence on this question. The subjects’ visual and auditory performances were evaluated using IVA-CPT, which provided a general score on an individual’s level of attention in visual and auditory domain, without sub-scores in this progress. Given the limitation of the evaluation tool, we cannot make any conclusion beyond the overall cognitive deficits in strabismic children. However, we believe that, once we have a consensus on strabismic-induced attentional deficit, we can further conduct research to reveal the mechanisms of this process.

At the same time, the results provided a general score on an individual’s level of attention in visual and auditory domain. **(Lines 346 to 348)**

It is important to assess which specific part in the cognitive process are delayed or distorted in strabismic children. **(Lines 352 to 353)**
